# Capturing the Cranio-Caudal Signature of a Turn with Inertial Measurement Systems: Methods, Parameters Robustness and Reliability

**DOI:** 10.3389/fbioe.2017.00051

**Published:** 2017-08-23

**Authors:** Karina Lebel, Hung Nguyen, Christian Duval, Réjean Plamondon, Patrick Boissy

**Affiliations:** ^1^Faculty of Medicine and Health Sciences, Orthopedic Service, Department of Surgery, Université de Sherbrooke, Sherbrooke, QC, Canada; ^2^Research Centre on Aging, Sherbrooke, QC, Canada; ^3^Département des Sciences de l’activité Physique, Université du Québec à Montréal, Montreal, QC, Canada; ^4^Centre de Recherche Institut Universitaire de Gériatrie de Montréal, Montreal, QC, Canada; ^5^Laboratoire Scribens, Département de génie Électrique, École Polytechnique de Montréal, Montréal, QC, Canada

**Keywords:** turn, deficit, signature, inertial motion capture, IMU, attitude and heading reference system

## Abstract

**Background:**

Turning is a challenging mobility task requiring coordination and postural stability. Optimal turning involves a cranio-caudal sequence (i.e., the head initiates the motion, followed by the trunk and the pelvis), which has been shown to be altered in patients with neurodegenerative diseases, such as Parkinson’s disease as well as in fallers and frails. Previous studies have suggested that the cranio-caudal sequence exhibits a specific signature corresponding to the adopted turn strategy. Currently, the assessment of cranio-caudal sequence is limited to biomechanical labs which use camera-based systems; however, there is a growing trend to assess human kinematics with wearable sensors, such as attitude and heading reference systems (AHRS), which enable recording of raw inertial signals (acceleration and angular velocity) from which the orientation of the platform is estimated. In order to enhance the comprehension of complex processes, such as turning, signal modeling can be performed.

**Aim:**

The current study investigates the use of a kinematic-based model, the sigma-lognormal model, to characterize the turn cranio-caudal signature as assessed with AHRS.

**Methods:**

Sixteen asymptomatic adults (mean age = 69.1 ± 7.5 years old) performed repeated 10-m Timed-Up-and-Go (TUG) with 180° turns, at varying speed. Head and trunk kinematics were assessed with AHRS positioned on each segments. Relative orientation of the head to the trunk was then computed for each trial and relative angular velocity profile was derived for the turn phase. Peak relative angle (variable) and relative velocity profiles modeled using a sigma-lognormal approach (variables: Neuromuscular command amplitudes and timing parameters) were used to extract and characterize the cranio-caudal signature of each individual during the turn phase.

**Results:**

The methodology has shown good ability to reconstruct the cranio-caudal signature (signal-to-noise median of 17.7). All variables were robust to speed variations (*p* > 0.124). Peak relative angle and commanded amplitudes demonstrated moderate to strong reliability (ICC between 0.640 and 0.808).

**Conclusion:**

The cranio-caudal signature assessed with the sigma-lognormal model appears to be a promising avenue to assess the efficiency of turns.

## Introduction

Functional mobility is a key component of the quality of life in older adults. Basic daily activities involve the execution of mobility tasks, such as walking, turning, standing up and sitting down. Turning, defined as a change in walking direction, is a specifically challenging mobility task which requires inter-limb coordination and postural stability to adequately follow the central nervous system instructions (Mancini et al., 2015a; Mellone et al., 2016). Turning must also be planned in advance to efficiently and safely process and execute the information leading to the modified trajectory (Patla et al., 1999). Deficits in postural transitions, such as turning, have been identified in frails (Galán-Mercant and Cuesta-Vargas, 2014) and persons with neurological deficits (Salarian et al., 2009; Mancini et al., 2015a) and are associated with a higher risk of falling (Mancini et al., 2016). It has also been shown that objective turn metrics (e.g., number of steps while turning) are able to identify individuals with mobility impairments better than traditional gait speed and clinical measures of mobility (Carpinella et al., 2007; Salarian et al., 2009; Zampieri et al., 2010; King et al., 2012; Spain et al., 2012). Consequently, studies have suggested an increased vulnerability to impairments during the turn compared to straight-line walking due to the complexity of the task and the neural systems involved (Herman et al., 2011). Recently, Hulbert et al. (2015) have suggested categorizing turning deficits into axial and perpendicular deficits, where perpendicular deficits relates to suboptimal movement in the limbs while axial deficits refers to inadequate movement of the head to trunk rotational axis. Perpendicular deficits would, therefore, include: an increased number of steps, related to the use of a compensatory strategy; a reduced step length, to maintain postural stability; and a modified turn strategy. Alternatively, axial deficits would include segment rigidity and segment rotation which would require the adoption of compensatory strategies, and segment coordination and timing, leading to overall uncoordinated movements. On a global scheme, all of these deficits may be viewed inter-related since full body control and coordination is required to safely execute a turn. Thus, Hulbert suggests that axial deficits may lead to altered control in perpendicular segments. If so, axial deficits may appear first and early assessment of such deficits may lead to better prevention.

In healthy individuals, it has been shown that efficient turning involves a cranio-caudal sequence of movement where the head initiates the motion, followed by the trunk and then the pelvis to efficiently steer the body into the desired new direction (Fuller et al., 2007; Hong et al., 2009). This sequence was shown to be altered in people with neurodegenerative disease and those who are recurrent fallers, exhibiting increased coupling of the segments (Ferrarin et al., 2006; Crenna et al., 2007; Hong et al., 2009; Wright et al., 2012; Spildooren et al., 2013). However, all of these observations were made in motion capture laboratories using camera-based stereophotogrammetric systems. Although powerful, such systems are expensive, complex to use, require a large dedicated space and have a constrained volume of acquisition (Zhou and Hu, 2008). As such, these systems are not well-adapted to clinical settings. To efficiently be used in a clinical context, a system must preferably be portable, configurable, relatively low-cost, easy to use, and output information must be easily interpreted from a clinical perspective (Ginsburg, 2005; Anderson et al., 2012; Gaudreault et al., 2012). Advances in wearable technology offer new possibilities for researchers and clinicians to assess mobility. Inertial measurement systems are among promising wearable sensors which have gathered an increasing interest in the past decade because of their portability, autonomy, acquisition frequency, and general form factor (size, and configuration) (Zhou and Hu, 2008; Horak et al., 2015). Inertial measurement systems include attitude and heading reference systems (AHRS), also referred to in the literature as magnetic and inertial measurement unit (MIMU), magnetic angular rate and gravity sensor, or Inertial and Meagnetic Unit (MIMU). AHRS are comprised of 3-axes accelerometers, gyroscopes, and magnetometers from which information is fed into a fusion algorithm to estimate the orientation of the module in a global reference frame based on gravity and magnetic North. Therefore, using multiple AHRS affixed on contiguous segments makes it possible to assess a person’s joints kinematics in different contexts. The diversity of sensors included within AHRS makes them good representative of commonly named movement monitors. This measurement system allows not only the quantity of activity performed to be monitored but also the quality of that motion through spatiotemporal gait and turn characteristics analysis as well as joint kinematics (Horak et al., 2015; Lebel et al., 2016).

Although multiple studies have used AHRS to assess mobility, the focus has always been on the raw sensors’ information (i.e., acceleration and/or segment angular velocity). Consequently, turn duration and turn speed were identified as useful measures to characterize age-related changes (Sheehan et al., 2014; Vervoort et al., 2016), identify recurrent fallers from non-fallers (Greene et al., 2010; Zakaria et al., 2015; Mancini et al., 2016), differentiate between healthy controls and early Parkinson’s disease patients (Salarian et al., 2009, 2010; Zampieri et al., 2010; El-Gohary et al., 2013; Mancini et al., 2015a), and frails (Galán-Mercant and Cuesta-Vargas, 2014). Although segment and joint orientation information may provide information on a person’s functional capabilities that is more easily interpreted, it is far less exploited. Validity studies have proven that the accuracy of the orientation data is sufficient for coarse clinical kinematic assessment (Ferrari et al., 2010; Zhang et al., 2013; Lebel et al., 2017). However, literature also clearly highlights possible variations in accuracy with changing magnetic environment (Roetenberg et al., 2007; Palermo et al., 2014; Schiefer et al., 2014; Yadav and Bleakley, 2014) while accuracy has also been shown to vary across joints and tasks (Palermo et al., 2014; Lebel et al., 2016). Recently, Lebel et al. (2017) suggested that this variation may be partly linked to an optimal region of operation for segment angular velocity. These uncertainties regarding orientation data accuracy may explain the current underutilization of such data. However, these limitations are mainly present in extremity kinematics, where segment velocities are higher and magnetic perturbations are more common (Palermo et al., 2014; Lebel et al., 2017). During a turn, both the head and the trunk’s angular velocity are within the optimal region of operation and magnetic perturbations can be assumed as minimal. Hence, the kinematic variation of the head relative to the trunk during a turn appears to be a good candidate to investigate the added value of AHRS orientation data analysis to derive meaningful clinical outcomes.

Traditionally, cranio-caudal sequence is assessed in biomechanics laboratories using camera-based stereophotogrammetric systems and analyzed in the temporal domain. Differences in temporal sequences are interpreted to be linked to different turning strategies. Such interpretations suggest that the cranio-caudal sequence exhibits a specific signature according to the adopted turn strategy. The so-called *movement signature* concept corresponds to the specific way (timing, force, amplitude, velocity) the movement is performed. Through signal modeling, the complex system involved in human movement can be reduced to a simpler form in order to better understand it. In this specific case, signal modeling is believed to provide insights into the mobility deficits. Human movement can be modeled using different paradigms which include, but are not limited to: equilibrium point models, minimization-based models, kinematic-based models and neural networks (Plamondon et al., 2014). Based on the Kinematics Theory, human movement can be seen as the cumulative response of an important number of biological systems (Plamondon, 1995a,b, 1998; Plamondon et al., 2003). Each system will produce a velocity vector from which their cumulative sum will, in the end, result in the movement of a segment. The motion can, therefore, be seen as the spatiotemporal representation of the energy induced on a specific body segment. The different systems involved in the planning and the execution of a specific task is controlled by the central nervous system. Therefore, assessment of human motion produced during a specific task can provide insights into the fundamentals of the motor control system (Wolpert et al., 1995). Analysis of the human motion through linear system modeling and an impulse response approach, therefore, seems to be a promising avenue for better characterization and early identification of motor control system deficits. Among those kinematic-based models are the delta- and sigma-lognormal models (Plamondon, 1995a,b; Djioua, 2007). These models rely on mathematical grounds to demonstrate that the lognormal function properly models the impulse response of the neuromuscular network in the case of rapid movements and can be seen as the optimal representation of the movement’s kinematics (Djioua and Plamondon, 2009). Their applications ranges from human motor control phenomena explanations and the factors affecting it (Plamondon and Alimi, 1997; Plamondon et al., 2013a) to scripted signature verification (Djioua and Plamondon, 2009; Woch and Plamondon, 2010; Woch et al., 2011; Plamondon et al., 2013b; Diaz et al., 2016) and detection of fine motor control problems (O’Reilly and Plamondon, 2011; O’Reilly et al., 2014) as well as applications to monitor the evolution of fine motor control in kindertgarden children (Duval et al., 2015; Rémi et al., 2015). Indeed, directional rapid movements produce an asymmetrical bell-shaped velocity profile. This can be represented by lognormal functions with characteristic parameters and can be related to the system commands and its ability to respond (command impulse delay, command magnitude, execution delay, and response time). However, can such model be used to analyze axial control specifically? Preliminary studies within the angular domain have shown that the wrist flexion and extension in monkeys could be fit very well with a delta-lognormal model (Plamondon, 1995a), but no extensive study has further explore the interest of using the Kinematic Theory for the analysis of angular movement control.

This study investigates the possibility of characterizing the turn cranio-caudal signature *via* a sigma-lognormal model using the head relative to the trunk velocity profile derived from the orientation data assessed with AHRS. Specifically, this paper aims at (i) presenting and illustrating the methods required for head-trunk signature recognition based on AHRS recording of motion and (ii) evaluating the robustness and the reliability of the identified cranio-caudal signature parameters.

## Materials and Methods

### Protocol and Instrument

**Figure 1:**
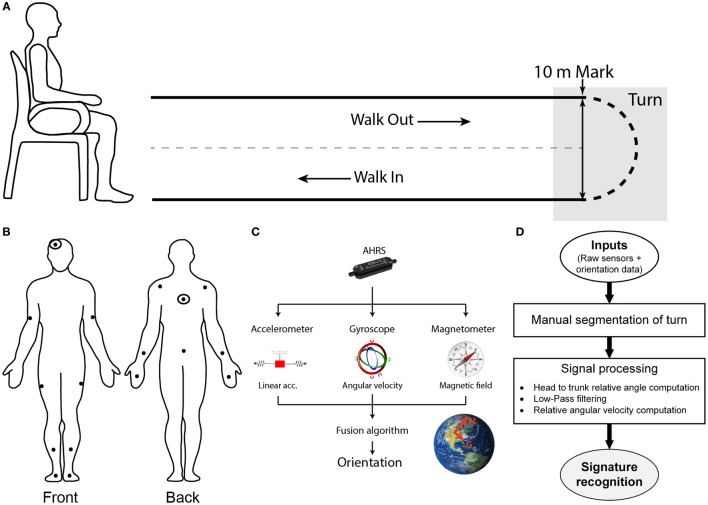
Setup, protocol, and methodology. **(A)** Spatial schematic of a 10-m Timed-Up-and-Go (TUG) task. Participants initiate the task sitting on a chair. Upon signal, the participant stands-up, walk out for 10 m, turn around when the 10-m mark is reached, walks back toward the chair and sits down. The turn portion of the TUG is targeted for the present study. **(B)** Participants are equipped with a suit comprised of sensors which position are illustrated this diagram. Signals from the double-marked sensors (head and trunk) were used for the signature recognition. **(C)** Sensors used are composed of 3-axis accelerometer, gyroscope, and magnetometer to measure linear acceleration, angular velocity, and magnetic field. All of the data are passed on to the fusion algorithm embedded in the sensor estimate the orientation of the module, expressed in an Inertial reference frame. **(D)** Global workflow of the algorithm to recognize the cranio-caudal signature of a turn.

The present study experimental protocol is based on the execution of a 10-m Timed-Up-and-Go (TUG). The TUG is a clinically recognized test to assess mobility and balance which combines basic mobility tasks (sit-to-stand, walk and turn) (Rehabilitation Institute of Chicago, 2010). Upon signal, the participant stands-up, walks out to the 10-m mark, turns around, and walks back to his initial seated position (Figure 1A).

To enable assessment of kinematics, participants are instrumented with the IGS-180 suit (Synertial Ltd., UK) containing 17 AHRS (OS3D, Inertial Labs, USA) as shown in Figure 1B. Each AHRS measures raw inertial signals (segment linear acceleration, angular velocity and magnetic fields) and derives the orientation of the module, and hence the orientation of the segment it is attached to, in a global reference frame (Figure 1C). A validity study performed on this system revealed an acceptable accuracy and an excellent agreement for both the head and trunk sensors when compared with an optoelectronic gold standard during a turn (Lebel et al., 2017). The IGS-180 enables acquisition of data (raw inertial data and orientation data) over its 17 sensors at 60 Hz. Sensor to body alignment, required to express the sensor movement into anatomical planes of reference, is performed with the participant standing in a neutral position (standing up, looking straight-ahead with palms facing their thighs) at the beginning of each trial.

### Signal Processing

Figure 1D gives an overview of the global workflow of the algorithm, including the signal processing. Trials are manually reviewed and segmented using the avatar in IGS-Bio, the application available with the IGS-180. Specifically, the procedure described below was followed to ensure systematic segmentation of the turns:

- visual identification of the point in time at which a misalignment between the head–trunk–pelvis axis appears;
- establishment of the beginning of the previous gait cycle (i.e., heel strike preceeding initial misalignment) → *Beginning of turn;*
- identification of the point in time at which the head-trunk-pelvis axis is realigned; and
- localization of the beginning of the next gait cycle (i.e., heel strike following realignment) → *End of turn;*

All trials were segmented by the same evaluator in order to avoid bias. Further signal processing is performed in Matlab v2015a (MathWorks, USA). For each trial, the relative orientation of the head to the trunk is computed and expressed in anatomical planes of reference. The resulting relative angle signals are then filtered using a fourth order low-pass Butterworth filter with a cutoff frequency of 1.5 Hz. The cutoff frequency was determined from a residual-based analysis of the relative orientation signal, using an acceptable threshold of 2° and was performed over repeated trials (Carbonneau et al., 2013). The residual threshold was based on the reported accuracy of orientation data obtained with the present system (Lebel et al., 2013, 2017). For each trial, the cutoff frequency that yielded the acceptable residual threshold was calculated. The final cutoff frequency was calculated from the mean and SD values obtained over repeated trials analysis to cover 95% of the cases. The resulting filtered angle profile was then transferred back into its quaternion form and used to compute the relative angular velocity profile.

Let us define

(1) $$\begin{array}{ll} \theta & \text{as the rotation angle and}\\ \vec{u}≜\text{u}=\left(u_{x}\text{i}+u_{y}\text{j}+u_{z}\text{k}\right) & \text{as the unit vector, expressed with the Cartesian axes }i,\text{j},\text{k} \end{array}$$

Then, the quaternion may be expressed as:

(2) $$\text{q}=\text{cos}\;\left(\frac{\theta }{2}\right)+\left(u_{x}\text{i}+u_{y}\text{j}+u_{z}\text{k}\right)\text{sin}\;\left(\frac{\theta }{2}\right)$$

(3) $$\underset{-}{\text{q}}=\;\left[\begin{array}{c} \text{cos}\;\left(\frac{\theta }{\text{2}}\right)\\ {\text{u}}_{\text{x}}\text{ sin}\;\left(\frac{\theta }{\text{2}}\right)\\ {\text{u}}_{\text{y}}\text{ sin}\;\left(\frac{\theta }{\text{2}}\right)\\ {\text{u}}_{\text{z}}\text{ sin}\;\left(\frac{\theta }{\text{2}}\right) \end{array}\right]$$

The angular velocity of the head relative to the trunk (ω) can then be determined by Eq. 4 (Rico-Martinez and Gallardo-Alvarado, 2000).

(4) ω=θ˙(t)u^(t)+u^. sin(θ(t))+u^(t)×u^.(1−cos(θ(t)))

The axial component of the angular velocity, corresponding to the axial velocity profile of the head relative to the trunk, is then available to be used for further signature analysis.

### Conceptual Framework and Parameters of Turn Signature

The optimal turn cranio-caudal sequence generates a change in relative angular orientation of the head to the trunk which segments are realigned upon completion of the transition. The turn cranio-caudal signature conceptual framework, therefore, has two main components: the analysis of the relative head to trunk maximum angle reached during the turn and the investigation of the relative angular velocity profile derived from it *via* the sigma-lognormal model approach.

#### Relative Angular Velocity Profile Analysis

According to the Kinematics Theory, the impulse response of the neuromuscular system (NMS) can be identified by analyzing the characteristics of the movement itself. If it is assumed that the NMS encompasses the motor cortex down to the muscles, all neuronal activities processed prior to the NMS consequently translates into a delay in the impulse command sent to the system. The NMS itself is made of multiple motor units which can be modeled as non-linear sub-systems organized in such a way that allows them to work efficiently (Plamondon, 1995a; Djioua, 2007). The impulse response of such linearized system follows an asymmetric positive bell-shaped curve described by a lognormal function. If one considers the control strategy of a movement from an energy point of view, the velocity of the end effector becomes the basic unit of the motion and should, therefore, follow a lognormal profile. Thus, Plamondon and his team proposed and validated the use of the sigma-lognormal model on the velocity profile to analyze the human motion during scripted signature (Plamondon, 1995a; Plamondon et al., 2003; Djioua, 2007; Djioua and Plamondon, 2009; O’Reilly and Plamondon, 2009; Javier et al., 2013).

**Figure 2:**
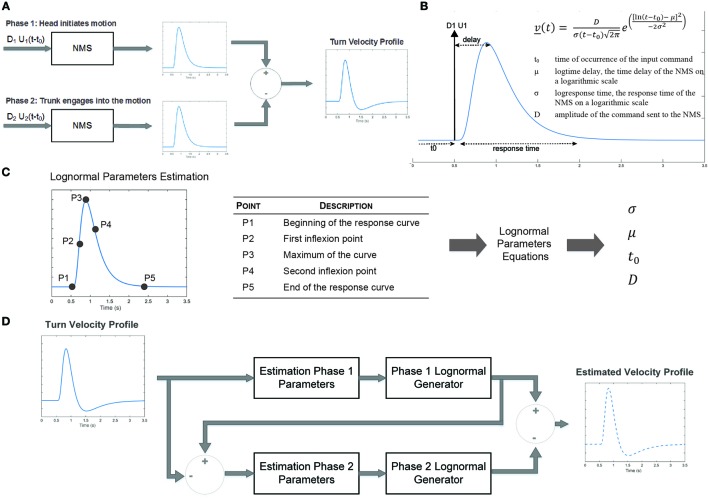
Sigma-log normal model conceptual framework. **(A)** Upon initiation of a turn, a first command is sent to the neuromuscular system (NMS) to initiate the head motion. A second command is sent to initiates the movement of the trunk. The difference of the NMS impulse responses generates the head to trunk velocity profile corresponding to the cranio-caudal signature. **(B)** The NMS impulse response is characterized by an asymmetric bell-shaped curved which can be characterized by the delay between command initiation and the median of the velocity as well as the response time. **(C)** Parameters of the sigma-lognormal profile can be estimated through the localization of specific points on the curve. **(D)** The sigma-lognormal model estimates the parameters of the two lognormal signal phases from which the velocity profile is estimated.

Here, we use the sigma-lognormal model to characterize the turn cranio-caudal signature. The two segments involved (head and trunk) can be seen as two NMSs, each one having its own lognormal impulse response. The output of each of these systems will, therefore, follow a lognormal profile for simple movements. In our study, we are interested in analyzing a more complex NMS, the head-trunk system, from which output can be seen as the vectorial summation of both basic systems outputs. Specifically, the cranio-caudal velocity profile can be decomposed into two phases corresponding to the moment the head initiates the turn, moving away from the trunk (phase 1) and the moment the trunk engages into the turn, closing the gap with the head (phase 2). We can, therefore, mathematically describe this complex system as the substraction of the two illustrated velocity profiles (Figure 2A; Eq. 5). The impulse response of the NMS is a lognormal (Plamondon et al., 2003), asymmetric bell-shaped curve (Figure 2B) from which the exact representation follows the equation in the insert and depends upon the magnitude of the commanded signal (D), the time occurrence of this command (t_0_), the execution delay (μ) and the response time (σ). The latter two were defined on a log scale. Indeed,

(5) $$\begin{array}{ll} \left|\vec{v}\left(t\right)\right| & =\;\left|\underset{i=1}{\overset{2}{\sum }}\;{\vec{v}}_{i}\;\left(t,t_{0i},\mu_{i}\sigma_{i}^{2}\right)\right|≅D_{h}\Lambda_{h}\;\left(t,t_{0h},\mu_{h}\sigma_{h}^{2}\right)-D_{T}\Lambda_{T}\;\left(t,t_{0T},\mu_{T}\sigma_{T}^{2}\right)\;;\text{and}\\ \Lambda_{i}\;\left(t,t_{0i},\mu_{i}\sigma_{i}^{2}\right) & =\frac{1}{\sigma_{i}\;\left(t-t_{0i}\right)\sqrt{2\pi }}e^{\left(\;\frac{{\left[\ln\left(t-t_{0i}\right)-\mu_{i}\right]}^{2}}{-2\sigma_{i}^{2}}\;\right)} \end{array}$$

where *t*_0_*_i_* is the time of occurrence of the *i*th input command; μ is the log time delay of the NMS, the time delay on a logarithmic scale; σ is the log response time of the NMS, the response time on a logarithmic scale; and D is the amplitude of the command sent to the NMS.

The lognormal equation parameters may be calculated using specific points of the velocity profile (Figure 2C) following equations, Eqs. 6–9 (Djioua, 2007; Djioua and Plamondon, 2009; O’Reilly and Plamondon, 2009).

(6) $$\frac{t_{P3}-t_{P1}}{t_{P5}-t_{P1}}=\frac{e^{-\sigma^{2}}-e^{-3\sigma }}{e^{3\sigma }-e^{-3\sigma }}\to \sigma$$

(7) $$\mu =\text{ln }\left(\frac{t_{P4}-t_{P2}}{e^{-\left(1.5\sigma^{2}-\sigma \sqrt{0.25\sigma^{2}+1}\right)}-e^{-\left(1.5\sigma^{2}+\sigma \sqrt{0.25\sigma^{2}+1}\right)}}\right)$$

(8) $$t_{0}=t_{P3}-e^{\mu }e^{-\sigma^{2}}$$

(9) $$D=\sqrt{2\pi }v_{P3}e^{\mu }\sigma e^{\left(\sigma^{4}∕2\sigma^{2}-\sigma^{2}\right)}$$

Indeed, from the velocity signal it is possible to identify the time at which the motion is initiated and terminated, the time at which the maximum velocity is reached as well as both inflection points. These points are first identified for phase 1 of the motion. The lognormal model parameters are then derived from these points and phase 1 response is estimated. A similar process is followed for phase 2, allowing a full reconstruction of the velocity signal (Figure 2D). From the estimated lognormal equation parameters, it is also possible to deduce further characteristics of the lognormal impulse response which could help interpret the NMS. The time delay $\left(\bar{t}\right)$, defined as the rapidity at which the system responds to the command, and the time response (s), corresponding to the time it takes the system to react and execute the movement, are defined by Eqs. 10 and 11, respectively (Plamondon et al., 2003).

(10) $$\begin{array}{ll} \bar{t} & =\int_{t_{0}}^{+\infty }t\Lambda \left(t,t_{0},\mu ,\sigma^{2}\right)\text{ dt}=t_{0}+e^{\mu +0.5\sigma^{2}} \end{array}$$

(11) $$\begin{array}{ll} s & =\sqrt{\int_{t_{0}}^{+\infty }t\Lambda \left(t,t_{0},\mu ,\sigma^{2}\right)\mathrm{dt}}=\sqrt{e^{2\mu +\sigma^{2}}\left(e^{\sigma^{2}}-1\right)}\\ & =\left(\bar{t}-t_{0}\right)\sqrt{\left(e^{\sigma^{2}}-1\right)} \end{array}$$

Finally, the quality of the reconstructed signature is evaluated using a signal-to-noise ratio (SNR) approach described in equation Eq. 12, as proposed by O’Reilly and Plamondon (2009).

(12) $$\text{SNR}=20\text{ log }\left(\frac{\int_{0}^{t_{\text{end}}}v^{2}\left(t\right)\mathrm{dt}}{\int_{0}^{t_{\text{end}}}{\left[v\left(t\right)-\hat{v}\left(t\right)\right]}^{2}\mathrm{dt}}\right)$$

In Eq. 12, *v* corresponds to the measured velocity profile, while $\hat{u}$ is the reconstructed or estimated profile.

#### Experimental Concept Overview

The complete set of metrics proposed for characterization of the turn cranio-caudal signature is summarized in Table 1. In order for these parameters to be of true interest, they must be robust to task velocity natural variation and be reliable.

**Table 1:** Turn cranio-caudal signature metrics.

Metric	Description
H2Tmax	Maximal head to trunk angle reached during turn
*D*_1_, *D*_2_	Amplitude of the commanded turn phase 1 and 2 signal
*t*_01_, *t*_02_	Time of occurrence of the commands (phase 1 and 2)
${\bar{\text{t}}}_{\text{1}},{\bar{\text{t}}}_{\text{2}}$	Time delay of the system impulse response (phase 1 and 2)
*s*_1_, *s*_2_	Neuromuscular system response time (phase 1 and 2)

### Detailed Experimental Protocol and Participants

The robustness and reliability of the proposed approach was tested on a sample of older adults. The project was approved by the Centre de Recherche de l’Institut Universitaire de Gériatrie de Montreal ethics board and participants provided written informed consent. Sixteen asymptomatic adults aged between 55 and 83 years old (mean age = 69.1 years, 50% female, height = 1.61 ± 0.08 m, weight = 63.2 ± 10.1 kg; BMI = 24.3 ± 3.2 kg/m^2^) participated in the study. Participants performed repeated 10-m TUGs equipped with the IGS-180, as explained in Section “Protocol and Instrument.” TUGs were executed both at normal and fast paces, each condition being repeated twice.

### Traditional Metrics

For comparison purposes, data were also analyzed using traditional metrics. As such, the accelerometer signal from the trunk AHRS was analyzed to determine the number of steps the participants took during the turn (Salarian et al., 2010). Analysis of the number of steps is based on a threshold on the acceleration measured by the trunk sensors. Validity of the method was assessed by visual comparison over five trials. Mean and max angular velocity during obtained during the turn was computed using the angular velocity data provided by the trunk AHRS’ gyroscope (Salarian et al., 2009, 2010; Mancini et al., 2015a).

### Data Analysis

For each trial, the introduced cranio-caudal signature metrics were calculated along with the traditional turn parameters.

A quality control process ensured that only the trials with a SNR greater than 10 dB were kept. The selected threshold is slightly lower than the generally accepted rule for SNR in controlled experiments (usually 15 dB), but this threshold was shown to be satisfying in this specific context. Indeed, this slightly more permissive SNR takes into account the complexity of the experiment and the possible sources for uncertainties such as the manual segmentation of the turn from the TUG task. The effects of velocity on the different metrics as well as their reliability were then analyzed. The robustness of the cranio-caudal turn signature metrics to natural task-related velocity variations and their reliability over repeated trials are important properties that need to be established before their validity can be further explored. All statistical analyses were performed using SPSS (v23.0.0 from IBM) and considered a significance level of 0.05.

#### Velocity Effect and Reliability

Each participant performed four TUGs (two at a normal pace, two at a fast pace). The effect of velocity on the metrics was, therefore, evaluated by taking the mean of each metric per participant and velocity and comparing them using a Wilcoxon signed-rank test. Reliability was assessed using a two-way random, absolute, average-measures intra-class correlation coefficient (Weir, 2005) performed on the repeated measurement of each metric [i.e., ICC(2,4) for absolute agreement]. The following guidelines were used for interpretation (Koo and Li, 2016):

- 0.00 ≤ ICC < 0.50 Poor reliability
- 0.51 ≤ ICC < 0.75 Moderate reliability
- 0.75 ≤ ICC < 0.90 Good reliability
- 0.91 ≤ ICC ≤ 1.00 Excellent reliability

## Results

**Figure 3:**
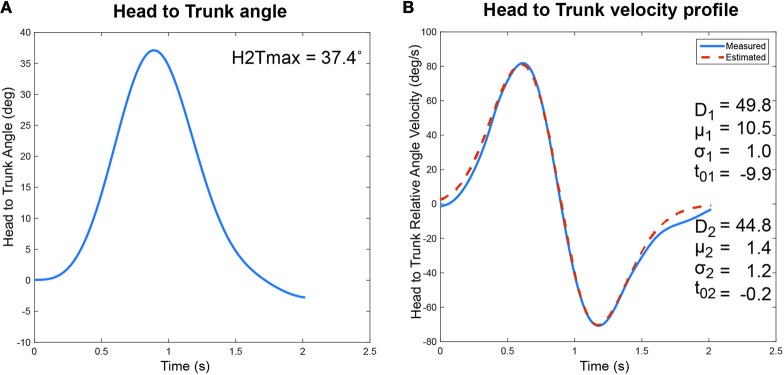
Cranio-Caudal Signature Determination. The proposed cranio-caudal signature approach is composed of both the analysis of the relative head to trunk angle achieved during the turn and the head to trunk relative angular velocity profile, modeled with the sigma-lognormal approach. **(A)** Change in head to trunk relative angle during a normal turn. The maximum angle reached is identified as a signature variable. **(B)** The blue curve illustrates the relative head to trunk angular velocity profile during the turn, as derived from the attitude and heading reference system measurement. The red dotted line illustrates the reconstructed profile, using the sigma-lognormal model. The parameters used to achieve the reconstruction are listed as inserts.

The ability of the sigma-lognormal model to estimate the cranio-caudal signature is shown in Figure 3. The left panel of this figure illustrates the variation in relative head to trunk angle captured during the turn for a healthy individual. The right panel corresponds to the relative head to trunk angular velocity profile for the same turn (blue curve—measured; red dotted curve—reconstructed profile using the sigma-lognormal approach). Analysis of the SNR revealed a median of 17.7 [14.6, 26.6], confirming the ability of the model to fit the data.

The robustness of the proposed parameters to velocity variations as well as their reliability shall now be verified.

### Velocity Effect

**Figure 4:**
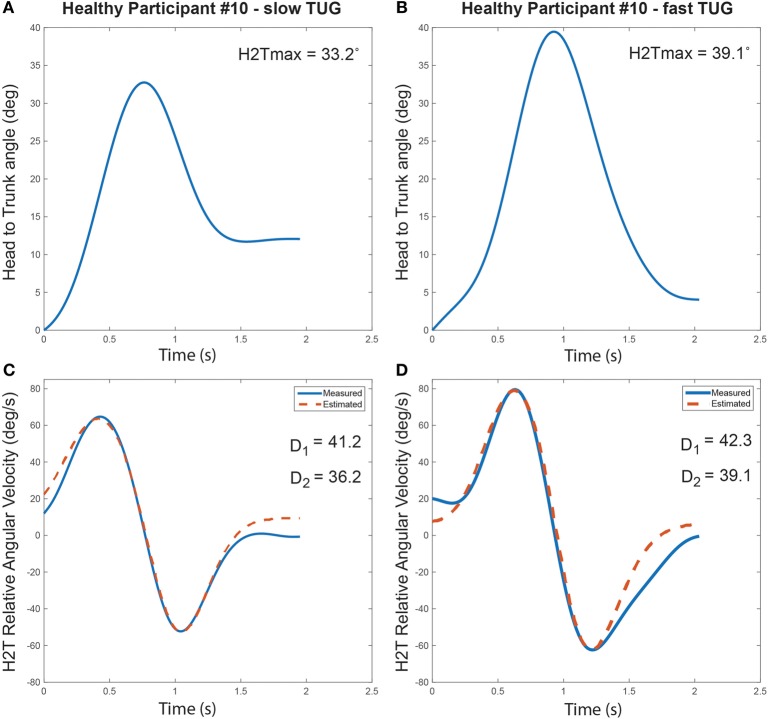
Turn cranio-caudal signature for a normal pace **(A,C)** and a fast pace Timed-Up-and-Go (TUG) **(B,D)**, executed by the same healthy participant. **(A,B)** Relative head to trunk angle variation captured during the turns. **(C,D)** Measured and estimated relative head to trunk angular velocity profile captured during the turns along with the computed signature parameters.

Normal pace TUGs were significantly slower than fast TUG (Normal pace TUG duration: 20.3 ± 2.8 s; fast pace TUG duration: 17.0 ± 1.7 s; *p* = 0.001). Figure 4 illustrates the turn’s cranio-caudal signature captured for the same healthy individual performing a normal pace and a fast pace TUG.

**Figure 5:**
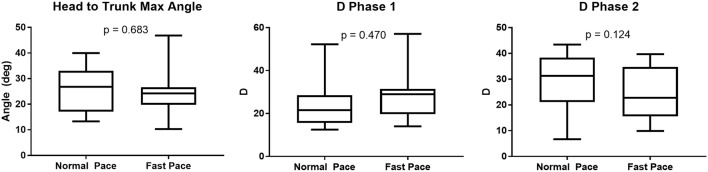
Turn signature metric dispersion per trial velocity.

**Figure 6:**
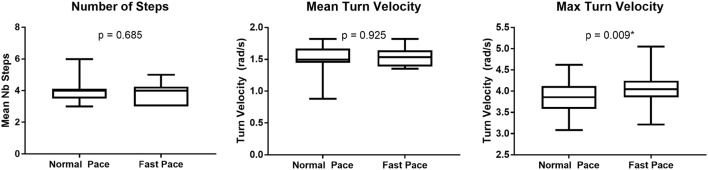
Tradition turn metric dispersion per trial velocity.

The dispersion of the cranio-caudal signature metrics (H2Tmax and D_1,2_) across participants is shown in Figure 5. The averaged peak head to trunk angle reached during the turn varied from 25.6° ± 8.9° for normal pace TUG to 24.5° ± 8.4° for fast pace trials, a difference not statistically significant (*p* = 0.683). The difference between the commanded amplitudes computed for normal pace versus fast pace were not statistically different (*D*_1_ normal pace: 24.8 ± 12.3, *D*_1_ fast pace: 28.5 ± 11.0, *p* = 0.470; *D*_2_ normal pace: 29.2 ± 11.0, *D*_2_ fast pace: 24.1 ± 10.1, *p* = 0.124). Similarly, the pace of the trials also did not have any significant effect on the timing parameters (*t*_01_ normal pace: −4.40 ± 6.30 s, *t*_01_ fast pace: −4.37 ± 5.56 s, *p* = 0.836; *t*_02_ normal pace: −7.0 ± 6.3 s, *t*_02_ fast pace: −4.5 ± 3.4 s, *p* = 0.198; ${\bar{t}}_{1}$ normal pace: 0.62 ± 0.15 s, ${\bar{t}}_{1}$ fast pace: 0.57 ± 0.21 s, *p* = 0.363; ${\bar{t}}_{2}$ normal pace: 1.34 ± 0.22 s, ${\bar{t}}_{2}$ fast pace: 1.17 ± 0.32 s, *p* = 0.158; *s*_1_ normal pace: 0.28 ± 0.09 s, *s*_1_ fast pace: 0.26 ± 0.06 s, *p* = 0.638; *s*_2_ normal pace: 0.23 ± 0.05 s, *s*_2_ fast pace: 0.22 ± 0.06 s, *p* = 0.198). For comparison purposes, Figure 6 illustrates the dispersion observed across participants for the traditional turn metrics. Both the number of steps (NbSteps normal pace: 3.9 ± 0.8, NbSteps fast pace: 3.9 ± 0.7, *p* = 0.685) and the mean turn velocity (turnvel_mean_ normal pace: 1.54 ± 0.25 rad/s, turnvel_mean_ fast pace: 1.53 ± 0.15 rad/s, *p* = 0.925) were not significantly affected by velocity. However, the maximum velocity was significantly different (turnvel_max_ normal pace: 3.83 ± 0.40 rad/s, turnvel_max_ fast pace: 4.08 ± 0.42 rad/s, *p* = 0.009).

### Reliability

Reliability was assessed for all repeated trials performed by the participants (i.e., normal and fast trials). Table 2 reports the ICC for each metric together with their 95% confidence intervals. Cranio-caudal signature metrics were shown to have a moderate to good reliability with ICCs, varying from 0.64 to 0.81. Furthermore, it was found that both traditional turn velocity metrics (mean and max turn velocity) had a moderate agreement while the number of steps revealed a poor reliability.

**Table 2:** Turn metrics reliability.

Metric		ICC	SEM
Cranio-caudal signature	H2Tmax	0.808 [0.422, 0.962]	3.9°
	*D*_1_	0.678 [−0.098, 0.939]	7.5
	*D*_2_	0.640 [−0.249, 0.933]	7.0
	${\bar{t}}_{1}$	−0.480 [−3.058, 0.693]	0.27 s
	${\bar{t}}_{2}$	0.781 [0.329, 0.957]	0.13 s
	*s*_1_	−0.045 [−1.818, 0.784]	0.11 s
	*s*_2_	0.538 [−0.707, 0.915]	0.04 s
	*t*_01_	−0.068 [−1.799, 0.664]	9.02 s
	*t*_02_	−0.216 [−2.823, 0.643]	9.25 s
Traditional metrics	NbSteps	0.242 [−2.179, 0.866]	0.8 step
	turnvel_mean_	0.607 [−0.397, 0.927]	0.13 rad/s
	turnvel_max_	0.682 [0.120, 0.935]	0.28 rad/s

## Discussion

The current study demonstrated for the first time that it is possible to successfully capture the cranio-caudal signature from the relative angular velocity profile deduced from the AHRS orientation data. In past studies, a cranio-caudal sequence was identified using camera-based stereophotogrammetric systems (Ferrarin et al., 2006; Crenna et al., 2007; Hong et al., 2009; Wright et al., 2012; Spildooren et al., 2013; Hulbert et al., 2015). These studies predominantly assessed the temporal sequence in which the segments (head, trunk and pelvis) are engaged in turning as well as the maximum angle reached by the head relative to the trunk and pelvis. In a study comparing recurrent fallers to non-fallers performing a 360° on-spot turning task, Wright et al. (2012) showed that all participants initiated the turn by rotating the head and that the extent of that head rotation is greater in non-fallers. Additionally, in a population with Parkinson’s disease, it was also shown that both the temporal cranio-caudal sequence as well as the maximum rotation of the head to the trunk are altered compared to controls, reflecting the so-called “en-bloc” strategy (Ferrarin et al., 2006; Crenna et al., 2007; Hong et al., 2009; Spildooren et al., 2013). Hence, it has been well demonstrated that the cranio-caudal sequence exhibited during the turn contains useful information. However, it is also documented that camera-based systems have restrictions (cost, required volume of operation, occlusions) which limit their use in a clinical settings (Zhou and Hu, 2008). Alternatively, inertial measurement systems have the portability required to be used outside laboratory settings, but the type of information provided by this system is different, and thus requires data to be analyzed differently. Orientation data, expressed in a global reference frame, allow us to measure the change in orientation of the head relative to the trunk. In this study, we investigated the possibility to capture and characterize the cranio-caudal signature from the orientation data provided by AHRS using a two-step process: First, the relative head to trunk angular profile is analyzed to assess the maximum angle reached. Then, the relative angular velocity profile of the head to the trunk is derived from that relative orientation information and investigated with the sigma-lognormal model. While orientation and inertial data (acceleration and angular velocity) can be used to directly characterize the turn, the choice to use a model is based on an assumption that this model will provide insights into the NMS which will help understand mobility deficits. The model has already been proven to be linked to the NMS in different situations, but had never been used on relative angular velocity. The combined analysis of the maximum relative head to trunk angle with a sigma-lognormal approach on the velocity profile of this joint, therefore, presents a promising avenue to enable cranio-caudal signature analysis with AHRS.

In order for the approach to be truly of interest, the signature metrics have to be reliable and robust to speed variations. Comparing the metrics computed during fast TUG to the ones computed for the TUG performed at normal pace has shown that velocity does not produce significant variations in the metrics. These results are in conjunction with Akram et al. (2010) who demonstrated, using a camera-based system, that the cranio-caudal timing sequence is robust to walking speed variations. Furthermore, the metrics have shown moderate to strong reliability over the four repeated trials. At this point, it is difficult to relate the results to other published work as this is, to our knowledge, the first time a similar approach has been used to characterize the cranio-caudal sequence. For comparison purposes, traditional metrics were also captured during each trial. These metrics (number of steps, mean turn velocity and max turn velocity) correspond to the current most popular metrics used in the literature to characterize the turn behavior using inertial measurement systems (Greene et al., 2010; Salarian et al., 2010; Zampieri et al., 2010; El-Gohary et al., 2013; Galán-Mercant and Cuesta-Vargas, 2014; Sheehan et al., 2014; Mancini et al., 2015b, 2016; Zakaria et al., 2015; Smith et al., 2016; Vervoort et al., 2016). Both the number of steps and the mean turn velocity were robust to a change in speed, but the maximum turn velocity was found to be significantly higher at fast pace TUG. According to Hulbert et al. (2015), the number of steps taken during a turn relates to the strategy adopted to perform that turn. The results from the current study illustrate that the turn strategy itself was not modified with TUG speed. In the literature, turn duration was identified as an objective biomarker of the ability of the neural control system to perform postural transitions (Horak and Mancini, 2013). Therefore, the observed increased maximum turn velocity with increasing TUG pace combined with the constant mean velocity can be interpreted as an adaptive strategy to maintain the same turn duration, denoting a good ability to change motor program among the participants. However, from these results, we must be cautious when interpreting a difference in maximum velocity to differentiate populations, as the extent of the difference may also be due to speed difference. If the instruction is not standardized (e.g., “perform the test as fast but safely as possible”), results of the maximum velocity may be biased. With respect to reliability, those traditional metrics performed poorer than the signature metrics as a result. The number of steps even showed poor reliability as assessed with an ICC. Previously, Salarian et al. (2010) had reported a strong agreement for that same metrics. The difference may be explained by the small variation between individuals within our sample. Indeed, the number of steps required to perform a 180° lacks variability in the current study as participants were all healthy elderly. Salarian et al. (2010) used both healthy controls and Parkinson’s disease patients to test for reliability, increasing the variability between individuals. In the near future, a test-retest reliability of cranio-caudal signature parameters could be re-evaluated using a similar approach to enable better comparison with the literature. The lack of variability between healthy individuals is a good thing when trying to differentiate two groups with clearly different behavior (e.g., Parkinson’s disease patients versus healthy controls). However, it raises concerns regarding the sensitivity to the change of such metric. The better reliability of the cranio-caudal signature metrics observed between individuals suggests a better resolution of the metrics, offering the potential to a better sensitivity to change. If true, such metrics could be useful to monitor changes in motor control with age or disease progression within individuals. One limit to this study is the fact that the proposed cranio-caudal signature methodology was directly validated using an inertial system which is known to have a certain inaccuracy. In a recent study, it was demonstrated that the segment of interest here had a mean root-mean-squared difference between 3.1° and 4.4° during a turn with peak values around 6°(Lebel et al., 2017). However, peak error will occur around maximum velocity which, in the case of the sigma-lognormal model, is defined by Eq. 13 below. The impact of this inaccuracy on timing parameters is minor as the reported agreement is good. As a result, inaccuracy in Vmax measurement could result in inaccuracy in the estimation of parameter D. However, recalling that the effect of the pace of the trial on D was shown to be not statistically significant across individuals, it can be assumed that the model is robust to the measurement inaccuracies:

(13) $$V_{\text{max}}=\frac{D}{\sigma ∕2\pi }e^{\left(-\mu +0.5\sigma^{2}\right)}.$$

Now that we have established the required methodology to derive the cranio-caudal signature based on AHRS data and verified the reliability of the metrics, there is a possibility of applying it to different populations to verify the sensitivity of the metrics.

The proposed algorithm allows for the characterization of the quality of a turn using AHRS in an innovative manner. It also demonstrates the power of orientation data assessed with AHRS. The full potential of such an approach will only be reached when combined with automatic recognition and segmentation of activities (Nguyen et al., 2015; Ayachi et al., 2016a,b). Additionally, this work also shows that the sigma-lognormal model can be used to fit the cranio-caudal signature. Although this model has been proven well-suited for rapid (Plamondon et al., 2014) and slow movements (Duval et al., 2015) in different situations, the movement of the head to the trunk during the turn is somewhat different and it was previously unclear if such a model could be applied here. The present results confirm this hypothesis. However, further validation of the model in this specific context of use would be beneficial in order to provide a deeper understanding of the parameters values in this particular framework.

## Conclusion

The present study has shown that cranio-caudal signature during the turn can be captured using AHRS and a sigma-lognormal model. Metrics deduced from the signature profile were shown to be robust to speed variations and reliable. Comparison with traditional turn metrics leads us to believe that the proposed approach is a promising avenue to enhance early deficits identification.

## Ethics Statement

Participants gave their informed consent following the procedure approved by the Centre de Recherche de l’Institut Universitaire de Gériatrie de Montreal ethics board.

## Author Contributions

KL developed the algorithm, designed the analysis, and drafted the manuscript. HN provided significant feedback on the analysis of the study and the manuscript. RP provided substantial feedback on the use of the Sigma-Lognormal model and its interpretation and reviewed the manuscript. CD conceived the experiment, helped in data interpretation, and reviewed the paper. PB helped in the conception of the algorithm, the interpretation of the data, and reviewed the analysis and the manuscript.

## Conflict of Interest Statement

The authors declare that the research was conducted in the absence of any commercial or financial relationships that could be construed as a potential conflict of interest.
